# A high-efficient and naked-eye visible CRISPR/Cas9 system in *Arabidopsis*

**DOI:** 10.1007/s00425-022-04060-5

**Published:** 2023-01-04

**Authors:** Wenwen Kong, Mingliang Wang, Lijuan Huang, Feiyan Wu, Jinyuan Tao, Beixin Mo, Yu Yu

**Affiliations:** Guangdong Provincial Key Laboratory for Plant Epigenetics, College of Life Sciences and Oceanography, Shenzhen University, Shenzhen, 518060 China

**Keywords:** CRISPR/Cas9, dsRED2, Genetic screening, *WUS* promoter

## Abstract

**Main conclusion:**

Introducing *35S*-*dsRED2* into the Cas9 vector which expresses naked-eye visible dsRED2 greatly facilitates the genetic screening, and the *WUS* promoter driving the Cas9 expression can improve editing efficiency in *Arabidopsis*.

**Abstract:**

CRISPR/Cas9-dependent genome editing has been applied to generate random insertions and deletions, targeted insertions or replacements, and precise base changes for both fundamental studies in many plant species and crop improvement. To simplify the screening procedure for target gene-edited transformants, we introduced a CaMV *35S*-driven *dsRED2* cassette (*35S-dsRED2*) into the Cas9 vector to express the naked-eye visible protein dsRED2, which can be observed under white light, greatly facilitated the genetic screening and reduced labor intensity without using any instrument. In addition, the *WUS* promoter was used to drive the expression of Cas9, which successfully improved the target genes editing efficiency and enabled the homozygous mutagenesis of two genes in T1 generation in *Arabidopsis*. Considering the conserved function and expression pattern of *WUS* across the plant species, this dsRED2-WUS/Cas9 system could also be used in many crops.

**Supplementary Information:**

The online version contains supplementary material available at 10.1007/s00425-022-04060-5.

## Introduction

The CRISPR/Cas (clustered regularly interspaced short palindromic repeats) (CRISPR-associated)-system is an RNA-mediated adaptive immune system in bacteria and archaea which provides defense against invasive genetic elements by cleaving the invader’s nucleic acid genome (Koonin et al. 2017). Based on its RNA-guided cleavage of DNA, the CRISPR system has been developed as a powerful and widely adopted genome engineering tool for targeted mutations in both animal and plant systems.

Nowadays, CRISPR/Cas9-dependent genome editing has been applied to generate random insertions and deletions, targeted insertions or replacements, and precise base changes for both fundamental studies in many plant species and crop improvement (Chen et al. 2019). Because Cas9 may continuously generate off-target mutations and make the genome unstable, it is almost impossible to conduct phenotypic characterization of an edited plant with the presence of Cas9 (Yu and Zhao 2019). However, it is laborious and money-time-consuming to remove the foreign DNA fragment by conventional genetic methods. Even mCherry and dsRED fluorescence proteins were previously used to screen the Cas9-free mutants in the T2 transgenic generation, all of them rely on expensive and precise microscopes (Gao et al. 2016; Aliaga-Franco et al. 2019; Yu and Zhao 2019). The low editing efficiency remains a significant obstacle to restricting its wide adoption in many plant species, especially in dicot plants. Previous studies have shown that Cas9 driven by the promoters of several genes, including the egg cell-specific *EC1.2* gene (Wang et al. 2015) and two cell division-specific *YAO* (*YAOZHE*, AT4G05410) and *CDC45* (*CELL DIVISION CYCLE 45*, AT3G25100) genes, improves the mutagenesis efficiency to around 8.3% ~ 30% in T1 transgenic plants (Feng et al. 2018), indicating that the spatiotemporal expression of Cas9 is one of the key factors. Inhibiting the essential genes in post-transcriptional gene silencing (PTGS) process, such as *RDR6* (*RNA-DEPENDENT RNA POLYMERASE 6*) and *AGO1* (*ARGONAUTE 1*), could further enhance the editing efficiency (Feng et al. 2018; Wang et al. 2019), but simultaneous mutations of PTGS genes and target genes may result in abnormal growth and defective propagation of plants, which greatly limits its broad application. In addition, although the *rdr6*/*ago1*-like phenotype can be used to screen T-DNA-free progenies, the screening of T1 transgenic plants still relies on antibiotics and/or herbicides. Therefore, it is important to develop tools to facilitate the genetic screening, reduce labor intensity as well as efficiently introduce precise target gene mutations in plants.

In this study, we introduced a CaMV *35S*-driven *dsRED2* cassette (*35S-dsRED2*) into the Cas9 vector to express dsRED2, a naked-eye visible protein (Jach et al. 2001; Wenck et al. 2003), which greatly facilitated and enabled the isolation of transgene-free plants with desired mutations of target genes in the T1 transformants in *Arabidopsis*. Meanwhile, the promoter of *WUS* (*WUSCHEL 1*, AT2G17950), an apical meristem-specific gene, was used to express Cas9, which achieved a higher editing efficiency than that of *pEC1.2::*Cas9 system.

## Materials and methods

### Plant growth and plant transformation

All Arabidopsis (*Arabidopsis thaliana*) plants used in this study are in the Columbia accession background and were grown under long photoperiod conditions (16-h light/8-h dark; white light; light intensity, 120 mmol m^−2^ s^−1^) at 22 °C.

For transformation, the target vectors were transformed into *Agrobacterium tumefaciens* strain *GV3101* and used for *Agrobacterium*-mediated floral dip*.*

### Plasmid constructs

The *dsRED2* gene were synthesized in Sangon Biotech (https://www.sangon.com) and ligated into the PCHF3 plasmid to generate the *35S-dsRED2* expression cassette. The *35S-dsRED2* was then cloned into the pHEE401 containing the *EC1.2::*Cas9 cassette to replace the *HYG* expression cassette to generate the dsRED2-EC1.2/Cas9 system. To generate the dsRED2-WUS/Cas9 and dsRED2-AS1/Cas9 system, the *EC1.2* promoter in dsRED2-EC1.2/Cas9 was replaced by the promoter of *WUS* and *AS1* (2 kb upstream of the CDS region), respectively. The plasmids which contained sgRNAs targeted to *GL1*, *TRY* and *CPC* were constructed as previously described with minor modifications. Briefly, two primers each contained one sgRNA were synthesized in Sangon Biotech, and the PCR fragments were cloned into the target plasmid using Homologous recombination Kit (C115-01, Vazyme). Primers used are listed in Supplemental Table S1.

### Plant genotyping

Genomic DNA was obtained from young leaves using CTAB extraction protocol. The gene’s target regions were amplified by PCR using specific primers. The PCR products were determined by Sanger sequencing. Primers used are listed in Supplemental Table S1.

### Western blot analysis

Approximately 0.5 g of 2-week-old seedlings were ground in liquid nitrogen and homogenized in protein extraction buffer containing 20 mM Tris–HCl (pH 7.5), 150 mM NaCl, 4 mM MgCl_2_, 75 mM ZnCl_2_, 0.1% Triton X-100, 1% glycerol, and EDTA-free protease inhibitor mixture (Roche). The cleared protein extracts were resolved on 12% SDS-PAGE gel. Immunoblot was performed using anti-RFP antibody with a dilution of 1/1000 (ab185921; abcam, Cambridge, UK) and anti-GAPDH antibody with a dilution of 1/2000 (60004-1-Ig; Proteintech, Rosemont, IL, USA). The protein signals were detected using the GE Healthcare Amersham TM ECLTM Prime Western Blotting Detection Reagent (RPN2232).

## Results and discussion

**Fig. 1:**
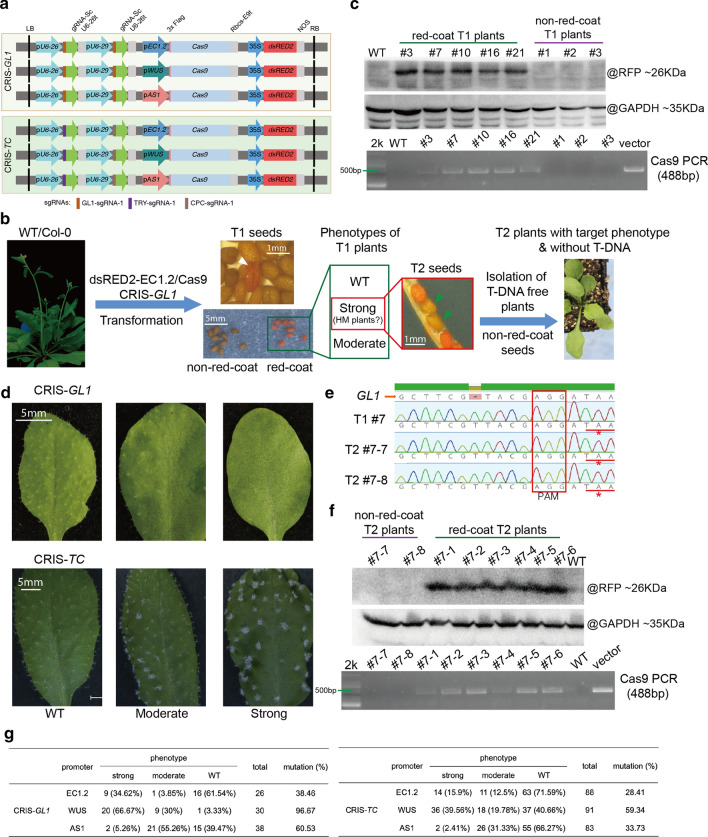
Determination of dsRED2-mediated screening of CRISPR/Cas9-edited transgene-free plants and analysis of the editing efficiencies of Cas9 driven by different promoters. **a** Physical map of the binary vectors generated in this study. **b** Workflow of the dsRED2-Cas9 system for screening the target gene-edited transformants in *Arabidopsis*. The white triangle indicates the redcoat seed. The green triangles indicate the non-redcoat-seed. **c** Detection of dsRED2 proteins and *Cas9* DNA fragments in the T1 redcoat-seed and non-redcoat-seed plants by Western blot and PCR, respectively. GAPDH was used as an internal reference. 2 k represents the 2000 bp DNA marker, the vector line indicates the Cas9 positive control. **d** Representative phenotypes of the strong and moderate mutants. **e** Sanger sequencing results of *GL1* loci for the T1 red-coat-seed and T2 non-red-coat-seed plants. The red stars indicate the early termination of *GL1* in the CRISPR edited plants. **f** Detection of dsRED2 proteins and *Cas9* DNA fragments in T2 redcoat-seed and non-redcoat-seed plants by Western blot and PCR, respectively. GAPDH was used as an internal reference. 2 k represents the 2000 bp DNA marker, the vector line indicates the Cas9 positive control. **g** Phenotype analysis of the transgenic plants generated by CRISPR/Cas9 with Cas9 driven by different promoters. Mutation (%) = (strong + moderate)/total

To simplify the screening procedure for target gene-edited transformants, we established a naked-eye visible reporter system by introducing the *35S-dsRED2* cassette into the *EC1.2* promoter-driven Cas9 vector (hereinafter referred to as dsRED2-EC1.2/Cas9) (Fig. 1a). *GLABRA1* (*GL1*), the component of trichome development, was chosen as the target gene to test the reliability of dsRED2-EC1.2/Cas9 system, and *gl1* mutant would exhibit glabrous leaves. One sgRNA targeting *GL1* was designed and cloned into both gRNA sites on the vector (Fig. 1a, Fig. S1), the dsRED2-EC1.2/Cas9-GL1 (CRIS-*GL1*) plasmid was then transformed to *Arabidopsis* Col-0 by *Agrobacterium*-mediated floral dip method. In the collected T1 seeds, many of them showed obvious redcoat (Fig. 1b), indicating the successful expression of dsRED2 in these transformants. After germination, the roots grown from the red-coated seeds also exhibited red color under white light, which was not observed in the untransformed Col-0 (Fig. S2). Next, both the red-coated and non-red-coated seeds were planted to check the expression of dsRED2. As expected, the dsRED2 protein was detected in the seedlings grown from red-coated seeds (red-seedcoat plant), but not in seedlings from non-red-coated seeds (non-red-seedcoat plant) (Fig. 1c). Consistently, Cas9 DNA fragment was only detected in red-seedcoat plants (Fig. 1c), suggesting that the visible red seedcoat could be a selection marker for genome wide insertion of foreign Cas9 fragment. Among the red-seedcoat plants which may undergo different types of mutagenesis in *GL1* gene, around 38.46% displayed varying degrees of defective trichome development, including WT (wildtype), moderate, and strong according to the severity of phenotypes (Fig. 1d, g). To examine whether the homozygous mutation occurs in T1 generation, we collected several individual plants with strong defect (no trichome throughout the plant) for sanger sequencing with a focus on *GL1* locus and successfully isolated the homozygous mutant *CRP-gl1 HM* (Fig. 1e). In the T2 population of *CRP-gl1 HM*, non-red-coat seeds representing dsRED2-free and Cas9-free were obtained, which performed same phenotype and mutation as their parents (Fig. 1b, e). In line with their morphological phenotype, neither dsRED2 protein nor Cas9 DNA fragment was detected in these plants (Fig. 1f). Collectively, these results demonstrated that the dsRED2-EC1.2/Cas9 system is a convenient, reliable and visual instrument-free tool for gene editing.

Parenchyma cells are more susceptible to the *Agrobacterium*-mediated infection than other cells. Previous reports have demonstrated that *WUS* and *AS1* are important regulators to maintain apical meristem function in *Arabidopsis* (Byrne et al. 2000; Wu et al. 2020). We wondered whether Cas9 driven by *WUS* and *AS1* promoters that specifically expressed in the apical parenchyma cells could improve the editing efficiency. To test this hypothesis, the promoters of *WUS* and *AS1* were cloned to replace *EC1.2* promoter in the dsRED2-EC1.2/Cas9-GL1 vector, respectively. Among the red-coated seeds obtained in T1 transformants of dsRED2-WUS/Cas9-GL1, about 66.67% seedlings displayed a strong uniform glabrous leaf phenotype, with an additional 30% showing the moderate phenotype (Fig. 1g, Fig. S3), indicating the greatly enhanced editing efficiency in *GL1* gene. For dsRED2-AS1/Cas9-GL1, although the total mutation rate (60.53%) was slightly increased compared to dsRED2-EC1.2/Cas9-GL1, the majority (55.26%) of T1 red-seedcoat plants only showed the moderate phenotype (Fig. 1g). In addition to *GL1* gene, *TRIPTYCHON* (*TRY*) and *CAPRICE* (*CPC*), another two components of trichrome development and the double mutant would have clustered trichomes, were chosen as targets to evaluate the editing efficiencies of these three systems. One sgRNA was designed close to the start codon of each gene and cloned into the same vector to simultaneously target both genes (CRIS-*TC*) (Fig. 1a, Fig. S1). Compared to dsRED2-EC1.2/Cas9-TC, which resulted in 15.9% strong and 12.5% moderate *try cpc* double mutants in T1 plants, dsRED2-WUS/Cas9-TC improved the rate to 39.56% and 19.78%, respectively. The homozygous *try cpc* double mutants were also identified (Fig. S4). For dsRED2-AS1/Cas9-TC, the total mutation rate was 33.73% and most double mutants exhibited moderate trichrome phenotype, which was similar to dsRED2-AS1/Cas9-GL1. Taken together, *WUS* promoter-driven Cas9 greatly enhanced the gene editing efficiency in *Arabidopsis*, which further proved that the spatiotemporal expression of Cas9 was important for its editing efficiency.

In conclusion, we established a high-efficient and convenient CRISPR/Cas9 gene editing system (dsRED2-WUS/Cas9) in plants. The expression of the naked-eye visible dsRED2 in transformants greatly facilitated the genetic screening and reduced labor intensity without using any instrument. In addition, the Cas9 driven by *WUS* promoter successfully improved the editing efficiency and enabled the homozygous mutagenesis of two genes in T1 generation. Moreover, no foreign DNA fragment affecting plant growth and development was introduced, which ensured the accurate observation of morphological phenotypes and the wide range of applications.

## Author contributions statement

Y.Y., W.K. and B.M. conceived the project. Y.Y., W.K., and B.M. designed the experiments. W.K., J.T. and M.W. generated the constructs. M.W., L.H., and F.W. performed the *Arabidopsis* transformation and analyzed the results. W.K. and Y.Y. wrote the manuscript. All authors read the manuscript and approved the final manuscript.

## Supplementary Information

Below is the link to the electronic supplementary material.

Supplementary file1 (DOCX 1957 KB)

## Data Availability

All data needed to evaluate the conclusions in the paper are present in the paper and/or the Supplementary Materials.
