# Supplementary material for: A high-efficient and naked-eye visible CRISPR/Cas9 system in Arabidopsis
Source: Planta. 2023 Jan 4;257(2):30. doi: 10.1007/s00425-022-04060-5 (PMC9810554; doi:10.1007/s00425-022-04060-5)
Supplement: Supplementary file 1 — Supplementary file1 (DOCX 1957 KB) [file 425_2022_4060_MOESM1_ESM.docx]

**Supplemental Information**

**Fig. S1 The sequence of WUS and AS1 promoter, as well as the synthetic sequence dsRED2-NOS sequence.**

**Fig. S2 The sgRNA sequences and positions targeting *GL1*, *TRIPTYCHON* (*TRY*) and *CAPRICE* (*CPC*), respectively. The sgRNAs are highlighted in red, and PAM sequences are indicated with gray background.**

**Fig. S3 The seedling roots of T1 red-coat seeds showed obvious red color compared with Col-0 under white light. Photoes were taken with 10-day old seedlings.**

**Fig. S4 Partial T1 seedlings used for phenotype analysis of CRIS-*GL1* and CRIS-*TC* for the Fig. 1g results. White scale bars indicate 5mm.**

**Fig. S5 Sanger sequencing results of *TRY* and *CPC* loci for the T1 red-coat-seed plants.**

**Table S1. Sequences of primers used in this study.**

**Supplemental Figures:**


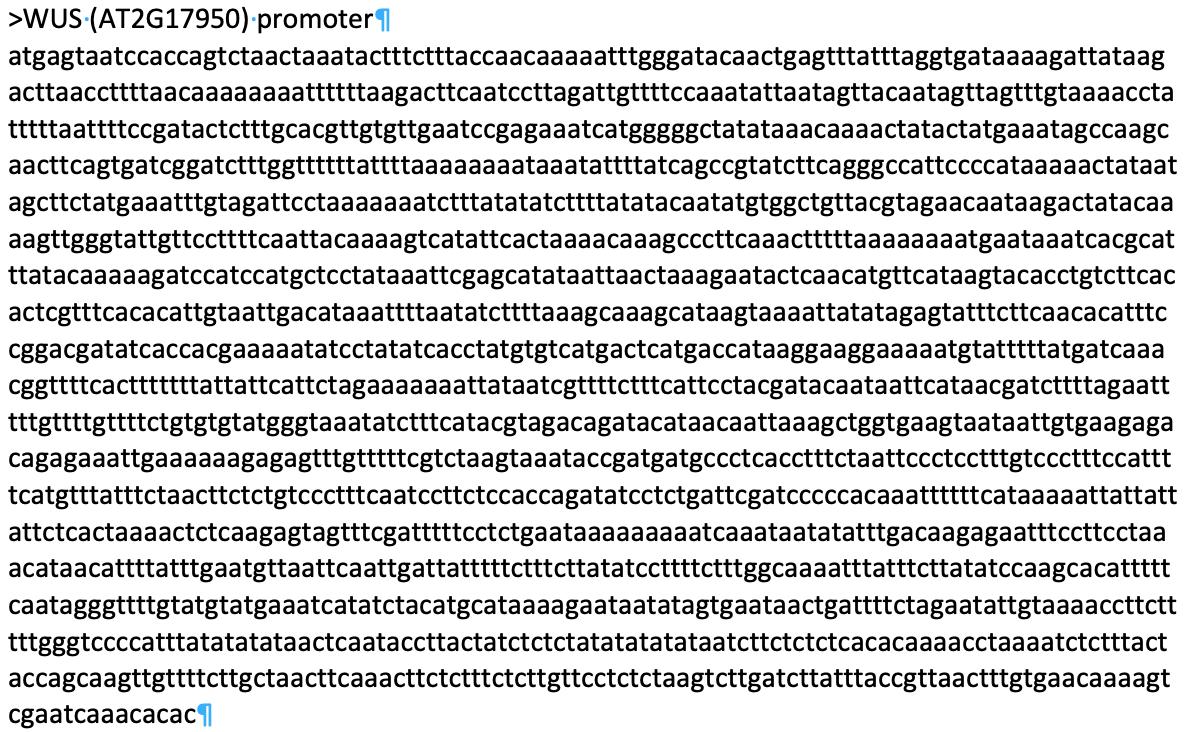


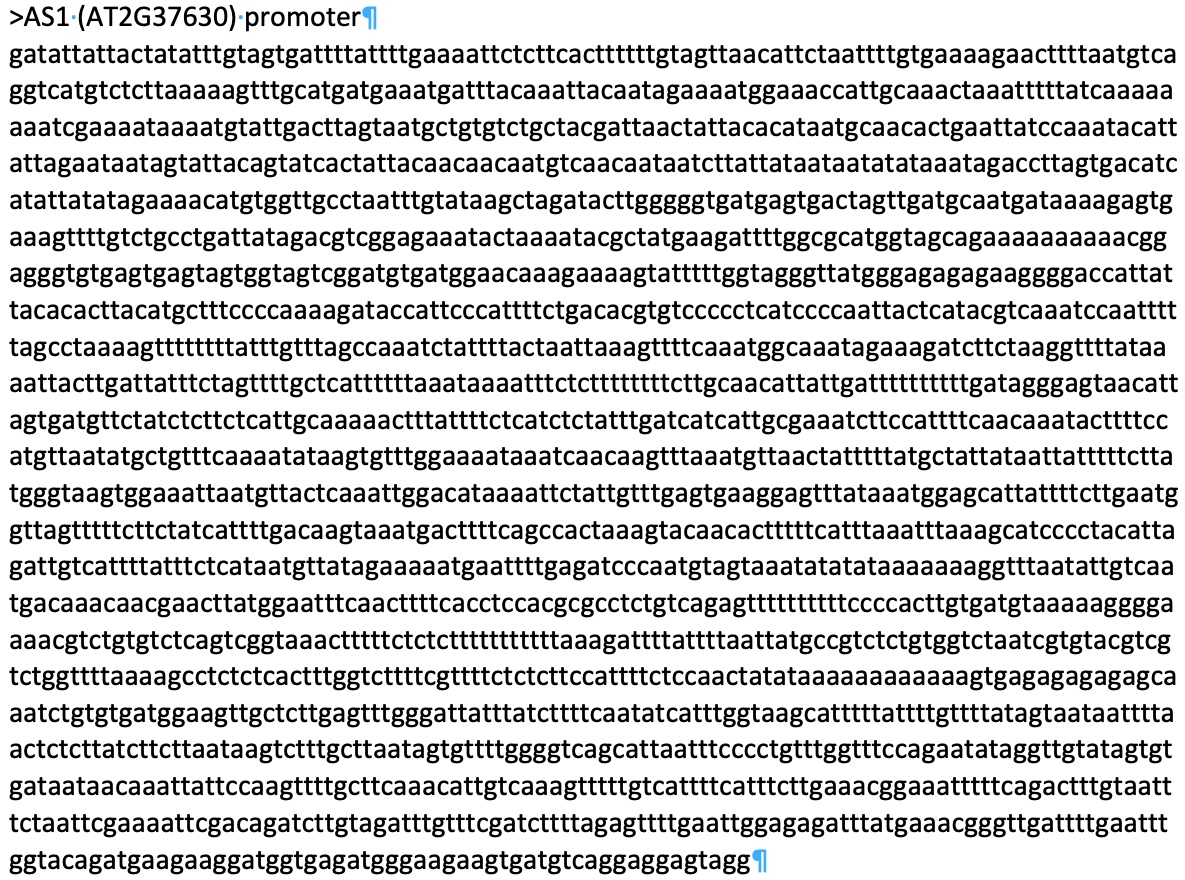


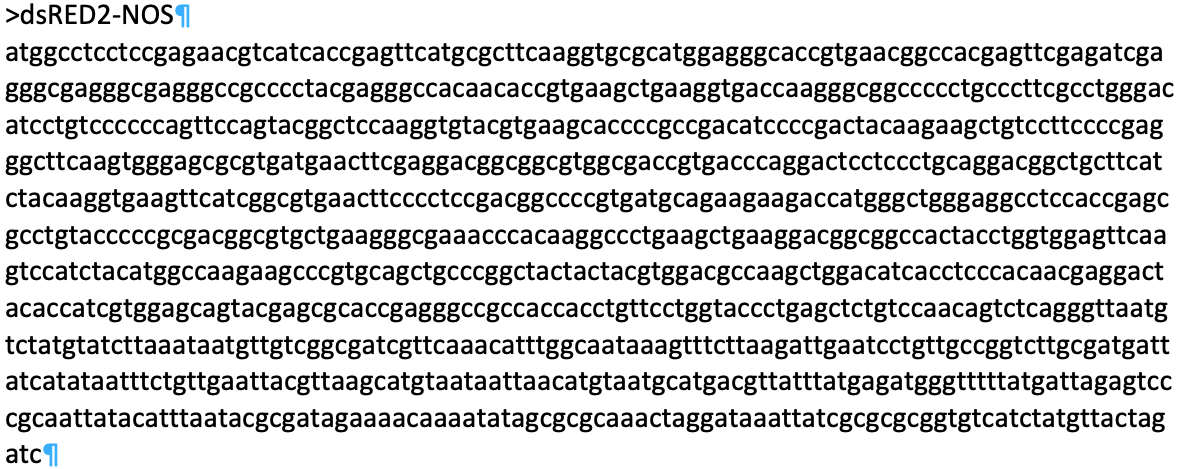


**Fig. S1** The sequence of *WUS* and *AS1* promoter, as well as the synthetic sequence *dsRED2-NOS* sequence.


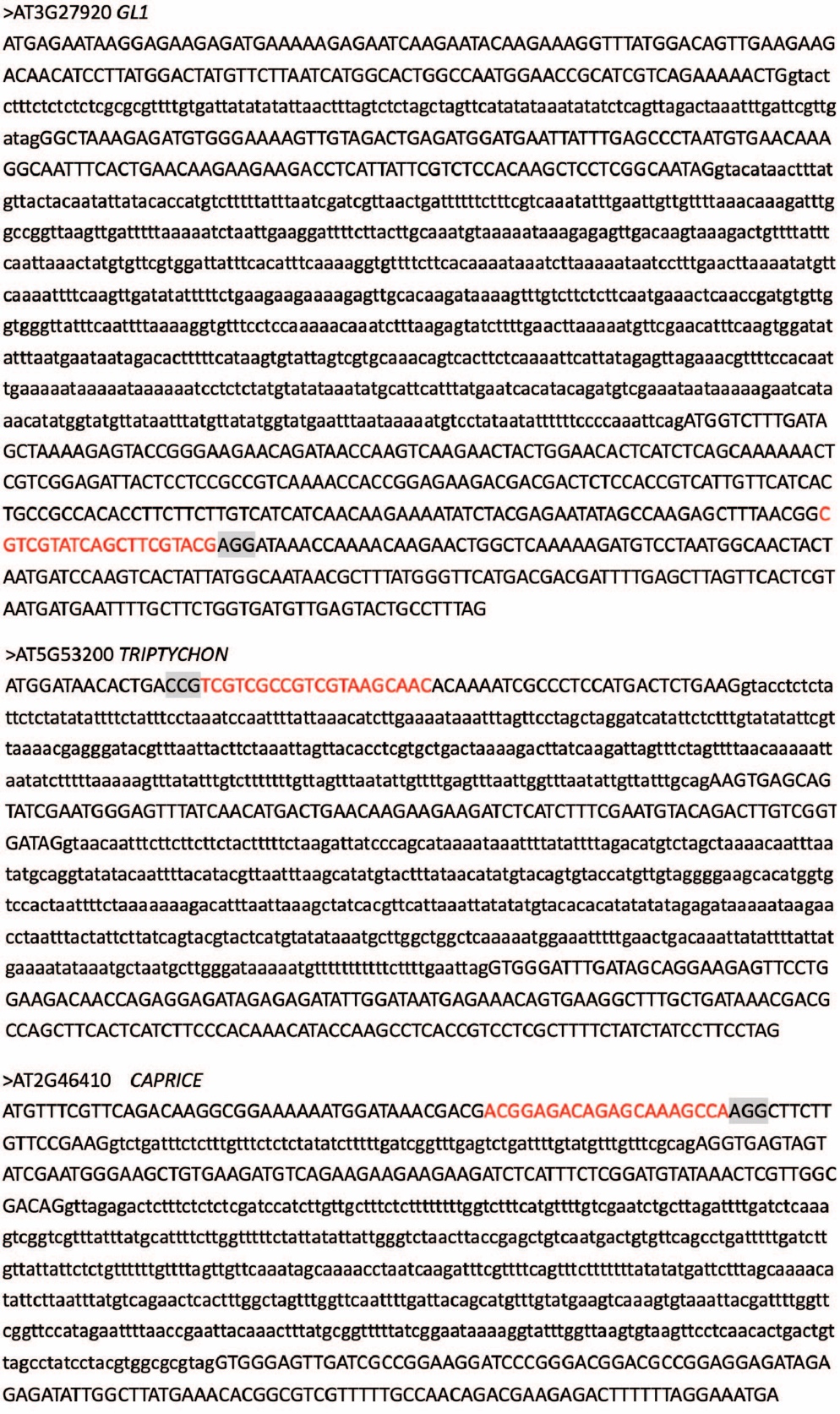


**Fig. S2** The sgRNA sequences and positions targeting *GL1*, *TRIPTYCHON* (*TRY*) and *CAPRICE* (*CPC*), respectively. The sgRNAs are highlighted in red, and PAM sequences are indicated with gray background.


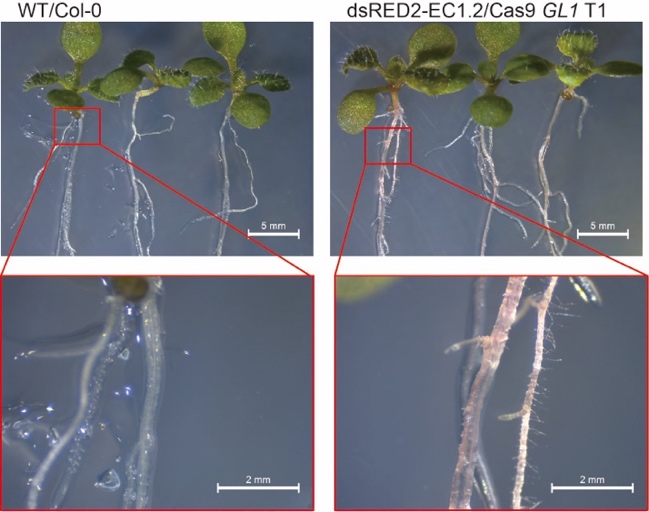


**Fig. S3** The seedling roots of T1 red-coat seeds showed obvious red color compared with Col-0 under white light. Photoes were taken with 10-day old seedlings.


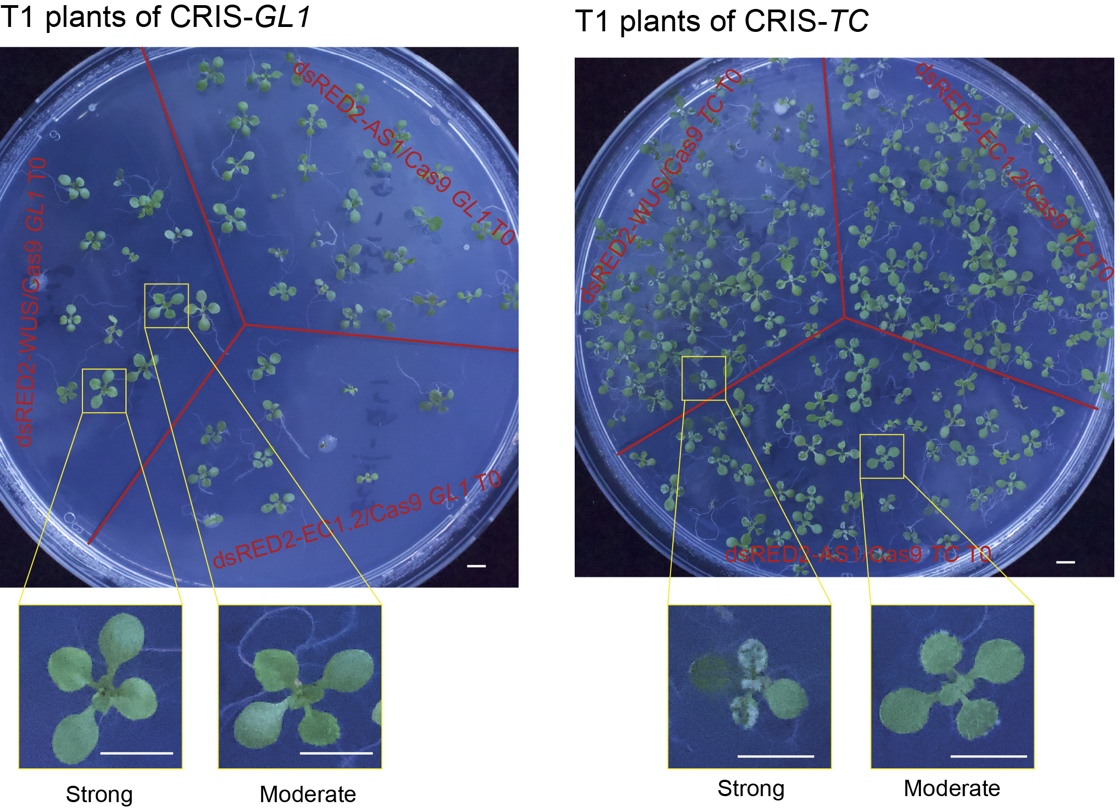


**Fig. S4** Partial T1 seedlings used for phenotype analysis of CRIS-*GL1* and CRIS-*TC* for the Fig. 1g results. White scale bars indicate 5mm.


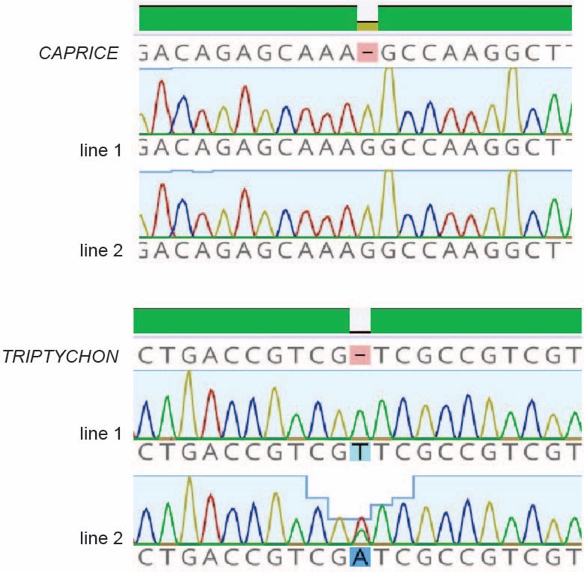


**Fig. S5** Sanger sequencing results of *TRY* and *CPC* loci for the T1 red-coat-seed plants of CRIS-*TC* transgenic line.

**Table S1.** Sequences of primers used in this study.

|  | Sequence (5'-3') |
| --- | --- |
| **Primers for Plasmid Construction** |  |
| GL1-CRP-F | GAGTCGAAGTAGTGATTGCGTCGTATCAGCTTCGTACGGTTTTAGAGCTAGAAATAG |
| GL1-CRP-R | CTATTTCTAGCTCTAAAACCGTACGAAGCTGATACGACGCAATCTCTTAGTCGACTC |
| TC-CRP-F | GAGTCGAAGTAGTGATTGACGGAGACAGAGCAAAGCCAGTTTTAGAGCTAGAAATAG |
| TC-CRP-R | CTATTTCTAGCTCTAAAACTCGTCGCCGTCGTAAGCAACCAATCTCTTAGTCGACTC |
| pWUS-F | CAATACCATGGTTATAATGAGTAATCCACCAGTCTAAC |
| pWUS-R | GTCCTTGTAATCCATGTGTGTTTGATTCGACTTTTGTTC |
| pAS1-F | CAATACCATGGTTATAGATATTATTACTATATTTGTAGTG |
| pAS1-R | CCTTGTAATCCATCTAGACCTACTCCTCCTGACATCAC |
| 35S-dsRED2-F | gattgacaacgaattcctagtagaaggtaattatccaagatg |
| 35S-dsRED2-R | gattacgaattccccgatctagtaacatagatgacaccgcg |
|  |  |
| **Primers for Plasmid sequencing** |  |
| U626-IDF | TGTCCCAGGATTAGAATGATTAGGC |
| U629-IDR | AGCCCTCTTCTTTCGATCCATCAAC |
|  |  |
| **Primers for genotype** |  |
| 401E-cas9-F | CCACGACGGGGATTACAAGGAC |
| 401E-CAS9-R | CGTGGTAGGCAACCTCGTCG |
|  |  |
| **Primers for mutation sequencing** |  |
| TRY-F | AAGGCCGTTCGTTGGACAT |
| TRY-R | GACACCATGTGCTTCCCCTA |
| CPC-F | CTACTATTAATCCTTCCCCTCGTG |
| CPC-R | CACAGTCATTGACAGCTCGG |
| GL1-F | CGTGCAAACAGTCACTTCTCA |
| GL1-R | GTACTCAACATCACCAGAAGC |
